# Pregnancy Zone Protein Serves as a Prognostic Marker and Favors Immune Infiltration in Lung Adenocarcinoma

**DOI:** 10.3390/biomedicines11071978

**Published:** 2023-07-13

**Authors:** Kehong Chen, Taihao Zheng, Cai Chen, Liangzhong Liu, Zhengjun Guo, Yuan Peng, Xiaoyue Zhang, Zhenzhou Yang

**Affiliations:** Department of Cancer Center, The Second Affiliated Hospital of Chongqing Medical University, Chongqing 400010, China; chenkehong@stu.cqmu.edu.cn (K.C.); zhengtaihao@cqmu.edu.cn (T.Z.); 2021110387@stu.cqmu.edu.cn (C.C.); liuliangzhong663@stu.cqmu.edu.cn (L.L.); guozhengjun@hospital.cqmu.edu.cn (Z.G.); pengyuan1127@cqmu.edu.cn (Y.P.)

**Keywords:** biomarker, lung adenocarcinoma, pregnancy zone protein, tumor infiltrating immune cells, macrophage, prognosis

## Abstract

Lung adenocarcinoma (LUAD) is a public enemy with a very high incidence and mortality rate, for which there is no specific detectable biomarker. Pregnancy zone protein (PZP) is an immune-related protein; however, the functions of PZP in LUAD are unclear. In this study, a series of bioinformatics methods, combined with immunohistochemistry (IHC), four-color multiplex fluorescence immunohistochemistry (mIHC), quantitative real-time PCR (qRT-PCR) and enzyme-linked immunosorbent assay (ELISA), were utilized to explore the prognostic value and potential role of PZP in LUAD. Our data revealed that PZP expression was markedly reduced in LUAD tissues, tightly correlated with clinical stage and could be an independent unfavorable prognostic factor. In addition, pathway analysis revealed that high expression of PZP in LUAD was mainly involved in immune-related molecules. Tumor immune infiltration analysis by CIBERSORT showed a significant correlation between PZP expression and several immune cell infiltrations, and IHC further confirmed a positive correlation with CD4+ T-cell infiltration and a negative correlation with CD68+ M0 macrophage infiltration. Furthermore, mIHC demonstrated that PZP expression gave rise to an increase in CD86+ M1 macrophages and a decrease in CD206+ M2 macrophages. Therefore, PZP can be used as a new biomarker for the prediction of prognosis and may be a promising immune-related molecular target for LUAD.

## 1. Introduction

Lung cancer has the highest mortality rate worldwide and is currently ranked as the second most commonly occurring tumor [1], 85% of which are non-small cell lung cancer (NSCLC). The common pathological type has changed from traditional squamous lung cancer to lung adenocarcinoma (LUAD) [2]. Despite numerous treatments for LUAD and improving technology, the survival rate for a period of five years remains no more than 20% [3]. In recent years, some potential biomarkers for LUAD have been proposed, such as Heat sock protein-90 Beta (HSP 90β) [4], Nucleophosmin 1 (NPM1) [5] and Aspartyl-TRNA Synthetase 2, Mitochondrial (DARS2) [6]. However, the occurrence and development of cancer is a complex biological process, and there is still an urgent need to develop new biomarkers.

PZP, a gene located on chromosome 12p13.31, is an α2 glycoprotein with a molecular weight of 359 kDa, and is structurally similar to α-2-macroglobulin, a member of the A2M family. This protein contains several protease cleavage sites, and upon binding to proteases, the conformation of this protein changes, thereby trapping the protease and limiting its activity [7]. The plasma concentration of PZP was originally found to increase significantly during pregnancy to inhibit fetal rejection [8], and decreased PZP could lead to recurrent and spontaneous abortions [9]. PZP was subsequently shown to achieve fetal protection from maternal immune system destruction [7]. Knockout of PZP may not prevent rejection of allogeneic heart transplants [10]. Human monocyte-macrophages mediate the binding, uptake, and degradation of the PZP–proteinase complex [11]. The above studies implied that PZP was functionally involved in the regulation of immunity. Recently, accumulating reports have provided evidence for a novel function of PZP in tumors. PZP is highly expressed in the serum of uterine leiomyoma, endometrial cancer and some ovarian tumors and its expression is decreased in the serum of malignant staphyloma and choriocarcinoma [8]. Moreover, it was found to be associated with low expression in breast cancer (BC) [12]. Knockout of PZP in breast cancer cells was found to promote BC progression by activating TGF-β/SMAD signaling [13]. Several studies have also shown low expression of PZP in hepatocellular carcinoma, and further exploration of its functions revealed that the PZP gene is highly methylated in hepatocellular carcinoma tissues and cells [14,15,16,17,18]. Unfortunately, the function of PZP in LUAD has not been adequately explored, although there are a few reports linking PZP to cancer.

Recently, the relationship between the tumor microenvironment (TME) and cancers has been an enthusiastically received topic of biomedical investigations, and increasing attention has been given to the close crosstalk between LUAD and the TME [19,20]. Immune cells infiltrating into the TME were observed to play a crucial role in the tumor immune response [21,22], and tumor infiltrating immune cells (TIICs) were positively related to better survival, highlighting the importance of TIICs in the prognosis for survival in patients with LUAD [23]. Nevertheless, the underlying function of PZP with TIICs remains unknown.

In this study, we combined public cancer databases with our clinical samples to investigate the prognostic value and conducted a tumor–immune interaction to research the potential role of PZP in LUAD. Our findings revealed the underlying function of PZP in predicting the risk of LUAD and the formation of an immuno-enhanced TME leading to tumor inhibition.

## 2. Materials and Methods

### 2.1. Data Acquisition

The TCGA and GTEx databases were utilized to retrieve gene mRNA expression levels and clinical parameters in cancer patients, comprising samples from the TCGA-LUAD cohort. From the raw data, PZP mRNA expression values, measured as transcripts per million (TPM), were extracted, and log2-transformed TPM values (log_2_[TPM+1]) were then computed [24]. To analyze the differences between normal and LUAD tissues, the Human Protein Atlas database (HPA) and Clinical Proteomic Tumor Analysis Consortium (CPTAC) were applied to assess the expression level of PZP protein. Additionally, survival curves were computed, based on the TCGA-LUAD cohort for patients’ overall survival (OS), and split into two groups: low-PZP expression (first third) and high-PZP expression (last third). To obtain the optimal prognostic model, both univariate and multivariate Cox regression analyses were employed. Furthermore, a nomogram was established, utilizing the R package rms to predict prognosis. The constructed nomogram was estimated using concordance index (C-index) and calibration charts, as reported by Longato [25]. Moreover, we constructed a receiver operating characteristic (ROC) curve, which helped in evaluating the diagnostic capabilities of PZP in LUAD.

### 2.2. Patient and Sample

A total of 110 pathologically confirmed LUAD paraffin-embedded (FFPE) tissues, along with paired para-cancer tissues located 4 cm away from the cancer tissues, were obtained through surgery performed at The Second Affiliated Hospital of Chongqing Medical University. No patients had accepted any form of therapy, such as radiotherapy, chemotherapy or targeted therapy, before surgery, and any patients with incomplete data were excluded from the research. The patients were all diagnosed with LUAD by a pair of experienced pathologists, and follow-up data was collected for all patients until January 2023, ranging from 1 to 60 months. The study group consisted of 51 male and 59 female patients aged between 48–78 years, with an average age of 65 years. The study received approval from the ethics committees of The Second Affiliated Hospital of Chongqing Medical University, and informed written consent was obtained from each patient, including any relevant details. All study methods were conducted in accordance with applicable guidelines and regulations.

### 2.3. Gene Set Enrichment Analysis

In order to determine the underlying pathways affected by PZP in LUAD, TCGA-LUAD data was utilized for GSEA analysis [26]. In this study, the normalize between arrays function of the R language limma package was used to standardize the expression profile of LUAD, and the standardized expression profile was obtained. According to the median PZP expression value in each LUAD sample, samples that exceeded the median threshold were placed in the high PZP expression, and samples less than the median were placed in the low PZP expression group.

Subsequently, GSEA v4 2.2 was utilized to identify potential biological processes or states affected by PZP. Specifically, Hallmarks (h.all.v7.5.1.symbols.gmt) was chosen as a predefined gene set for analysis to clarify specific processes. For each analysis, the value of the number of permutations was specifically designated as 1000, and the permutation type was carefully chosen as “phenotype”. All remaining parameters were left at their default values. In order to assess significant pathways, the statistical value was defined as nominal *p* < 0.05, and the threshold for the false discovery rate (FDR) was set to *p* < 0.25. The results were sorted by normalized enrichment scores (NESs).

### 2.4. Evaluation of TIICs

To analyze how PZP expression affects the immune microenvironment of the tumor, the subpopulations of 22 immune cell types infiltrating LUAD were estimated using CIBERSORT, a web-based tool that employs a deconvolution algorithm utilizing high-throughput sequencing data to measure immune cell composition [27]. Upon sorting the dataset, it was subsequently uploaded to the CIBERSORT portal, where the algorithm was executed using the immune cell subtype signature and 1000 permutations performed. A violin plot was generated, based on these results, with differences being tested by the Wilcoxon test. Results with a *p*-value less than 0.05 were used to determine statistical significance. Additionally, to visualize the interrelationships among the 22 distinct kinds of TIICs, a correlation heatmap was generated.

### 2.5. Immunohistochemistry (IHC) Staining

The paraffin slices were incubated upright for 4 h and dewaxed with xylene and alcohol of different concentrations (100, 95, 80, 70 and 50%), using EDTA repair buffer (pH = 9.0) for 15 min at room temperature (RT). A three-step immunohistochemistry rabbit SP kit (ZSGB-BIO, SP9001, Beijing, China) was used in this experiment. The endogenous peroxidase of the sample was inactivated after 15 min of incubation at 3% H_2_O_2_, and anti-PZP (Abcam, ab233166, Cambridge, UK, dilution ratio = 1:200), anti-CD68 (Cell Signaling Technology, #76437, Danvers, MA, USA, dilution ratio = 1:400), and anti-CD4 (Abcam, ab133616, UK, dilution ratio = 1:100) antibodies were added to the paraffin section after serum blocking for 1 h. After incubation at 4 °C for one night, the biotin-labeled goat anti-rabbit IgG polymer and streptomycin antibiotic-peroxidase were dropped onto the section for 15 min, respectively, at RT. DAB chromogen (Abcam, ab64238, Cambridge, UK) was used for visualization purposes. After staining with hematoxylin, the sections were subjected to dehydration, and subsequently mounted in neutral gum, and examined utilizing a bright field microscope from Olympus (Tokyo, Japan).

To calculate the H-SCORE for PZP staining, the proportion of positive cells was evaluated along with the integrated staining intensity in five randomly selected fields (magnification, ×200). The formula for PZP scoring was determined to be the multiplication of the proportion of positive cells by the staining intensity score and then multiplying by 100. The scoring of staining intensity was carried out according to the following grading system: 0, representing a lack of staining; 1, indicating weak staining; 2, denoting moderate staining; and 3, reflecting strong staining. Additionally, to assess the extent of infiltrating CD68+ cells and CD4+ cells in LUAD samples, cell counts were performed utilizing FIJI software (NIH, Bethesda, MD, USA, Version 2.1.0) on five randomly selected fields (magnification, ×200). All the above scores and cell counts were scored independently by two pathologists with an error of no more than 10%. If there was any difference, a third pathologist scored the results.

### 2.6. Cell Culture

The American Type Culture Collection (ATCC, Manassas, VA, USA) provided the following cell lines: H1650, PC-9, HCC827, A549 and BEAS-2B. H1650, PC-9, and HCC827 cell lines were cultivated in RPMI 1640 medium supplemented with 10% fetal bovine serum (FBS, Gibco, Franklin Lakes, NJ, USA) and 1% antibiotics. A549 and BEAS-2B cell lines were maintained in DMEM/F12 medium enriched with 10% FBS and 1% antibiotics. Passaging of cells was carried out every 2–3 days, and a growth environment of 37 °C and 5% carbon dioxide in a humidified incubator was maintained for the cells.

### 2.7. Quantitative Real-Time PCR (qRT-PCR)

The Quick RNA Extraction Kit (AG, AG21023, Changsha, China) was employed to extract total RNA from cells. The procedures were carried out in accordance with the instructions provided by the manufacturer. For reverse transcription, 1 µg of RNA was utilized along with the reverse transcription kit (AG, AG11728, Changsha, China), which included gDNA Clean in a 20 µL reaction. The PCR primers used for PZP and GAPDH were as described in Table S1. The PCR Kit (AG, AG11701, Changsha, China) was employed for performing qRT-PCR. Each 20 µL reaction comprised approximately 100 ng of cDNA, 0.4 µmol of each forward and reverse primer, and a volume of 10 µL, specifically 2 × Premix was used. Normalization of the expression data to the geometric mean of the internal reference gene GAPDH was performed to control expression level variability. The resulting expression levels were calculated as 2^−∆CT^. The PCR amplification was performed in triplicate.

### 2.8. Enzyme-Linked Immunosorbent Assay (ELISA)

The collected supernatant derived from PC-9, H1650, HCC827, A549, and BEAS-2B cell lines were used for ELISA analysis. One hundred microliters of supernatant were taken for each cell line and PZP levels were assessed using a commercial kit (Cloud-Clone Corp., #SEG324Hu, Wuhan, China). The statistical comparisons were conducted using one-way ANOVA with Dunnett’s multiple comparison test. The experiments were performed in triplicate.

### 2.9. Multiplex Immunohistochemistry (mIHC) Staining

A total of 40 tissues, including 20 cases of high PZP expression and 20 cases of low PZP expression, were selected for mIHC staining, according to the methods described by QIAO et al. [28] with minor modifications. In brief, slices were incubated upright for 4 h, dewaxed with xylene and alcohol (95%), and subjected to antigen retrieval using EDTA repair buffer (pH = 8.0, CST, #14747, Danvers, MA, USA) for 15 min. Quenching was performed with 3% H_2_O_2_ for 10 min. Anti-PZP (Abcam, ab233166, Cambridge, UK, dilution ratio = 1:200), anti-CD86 (CST, #91882, Danvers, MA, USA, dilution ratio = 1:200), and anti-CD206 (CST, #24595, dilution ratio = 1:600) antibodies were added to paraffin sections after serum blocking for 30 min, cultured in a wet cabinet for 60 min, added to HRP rabbit (CST, #8114, Danvers, MA, USA) and cultured in a wet cabinet for 30 min. Then, the tyramine signal was amplified using a four-color multi-labeling kit. Whole tissue scanning was performed using Pannoramic DESK (3D HISTECH, Budapest, Hungary) and FIJI software (NIH, Bethesda, MD, USA, Version 2.1.0) was utilized for the analysis of the images. The CD86+ and CD206+ cells were counted in five different fields using FIJI.

### 2.10. Statistical Analysis

For statistical analyses, GraphPad Prism 8 and R software (Version 3.6.2) were utilized. A Student’s *t*-test was employed to perform the comparison of differences between the two groups. Differences in clinicopathologic characteristics in patients with LUAD were evaluated utilizing the chi-square test. The effect of PZP expression on the overall survival (OS) of patients suffering from LUAD was conducted through a Kaplan–Meier survival analysis. Using a Cox proportional hazards regression model, the evaluation of the correlation between PZP expression, other clinical parameters and OS was performed. Statistical significance was attributed to PZP expression (*p* < 0.05).

## 3. Results

### 3.1. A Notable Decrease in PZP Expression Was Observed in Both Databases and Clinical Samples

**Figure 1:**
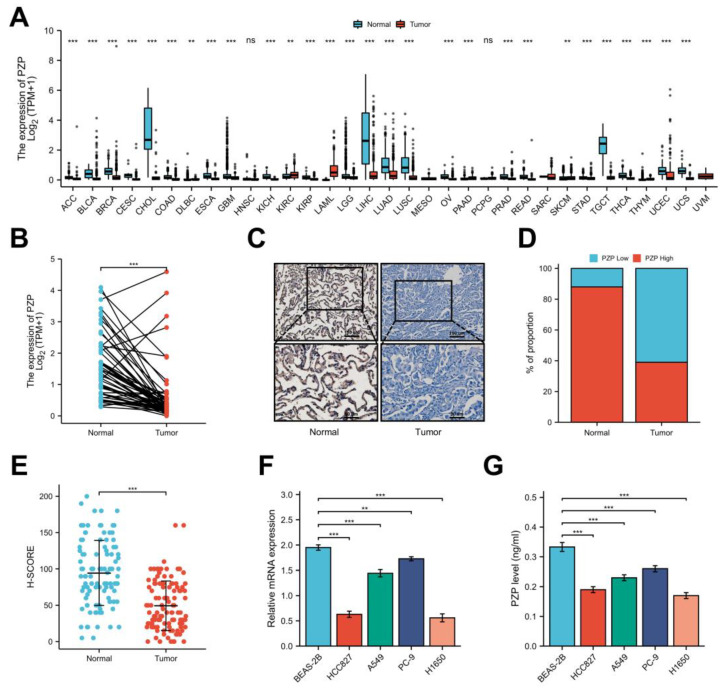
PZP was expressed at low levels in lung adenocarcinoma tumors. (**A**) PZP mRNA expression levels in different tumor samples, based on public databases. (**B**) The PZP mRNA expression disparity between normal tissues and LUAD samples was presented in paired samples. (**C**) The expression of the PZP protein in human LUAD and paired para-cancerous tissues was evaluated using IHC analysis. (**D**) The proportion of high-PZP and low-PZP protein levels in LUAD and adjacent normal tissue specimens. (**E**) The H-SCORE of the PZP protein is displayed. (**F**) The qRT-PCR detected the PZP mRNA level in cell lines. (**G**) ELISA of secreted PZP protein abundance in supernatants from cell lines. ns *p* ≥ 0.05 (no significant); ** *p* < 0.01; *** *p* < 0.001.

PZP mRNA expression in human pan-cancers was first analyzed using the TCGA and GTEx databases. Lower expression of PZP was observed in most tumors compared with normal tissues, such as cholangiocarcinoma (CHOL), lung squamous cell carcinoma (LUSC), urothelial bladder carcinoma (BLCA), testicular germ cell tumor (TGCT), hepatocellular carcinoma (LIHC) and breast invasive carcinoma (BRCA) (Figure 1A). Consistently, we also found that, compared with normal lung tissue (n = 347), PZP mRNA in LUAD (n = 598) was strikingly reduced, as can be observed in Figure 1A. The same trend was found in paired normal and tumor tissues (n = 59) (Student’s *t*-test *p* < 0.001, Figure 1B). Subsequently, we examined the PZP protein expression in normal and tumor tissues using the HPA and CPTAC databases, and the results were consistent with those of mRNA expression (Student’s *t*-test *p* < 0.001, Figure S1A,B). To validate the findings obtained from the public cancer databases, we performed IHC analysis of PZP expression in a total of 110 LUAD cases and corresponding para-cancerous tissues. The expression of PZP was located in the cytoplasm, secreted out of the cell and stained in brownish yellow granules (Figure 1C). Concurrently, the observations revealed that the positivity rate of PZP protein was notably lower in LUAD (39% vs. 61%) than in para-cancerous normal tissues (88% vs. 12%) (Figure 1D). H-SCORE was employed for PZP staining, according to the methods described by Heynemann [29]. As a result, significantly lower PZP staining was observed in tumor tissues (Student’s *t*-test *p* < 0.001, Figure 1E). Moreover, as shown in Figure 1F, the PZP mRNA level in tumor cell lines was obviously lower compared to the normal human bronchoalveolar cell line, BEAS-2B. The ELISA results revealed that the secreted PZP protein abundance in supernatants from LUAD cell lines was inferred to that in BEAS-2B cells (Figure 1G). These findings illustrated that PZP expression was downregulated and indicated that PZP contributes to the progression of LUAD.

### 3.2. PZP Has a High Diagnostic Value and Can Be Used as an Independent Prognostic Predictor

**Figure 2:**
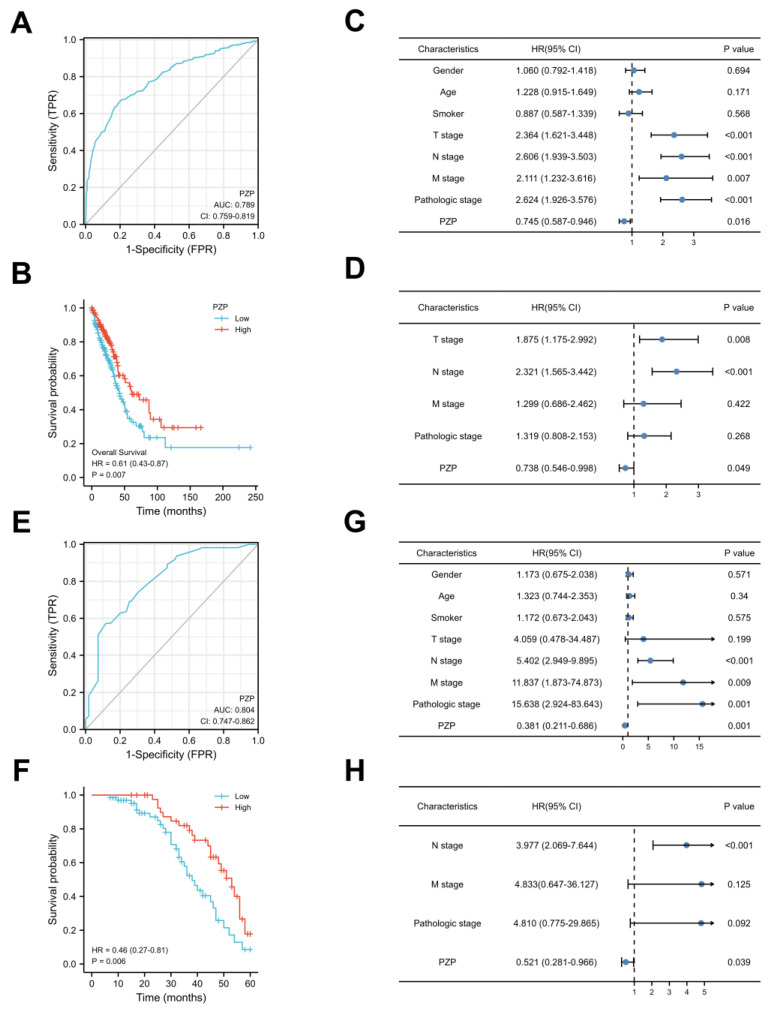
ROC curve and prognostic analyses in databases and clinical samples of LUAD. (**A**) ROC curve for PZP mRNA expression in the public databases. (**B**) The OS in TCGA–LUAD exhibited correlations with PZP mRNA expression. (**C**) Univariable and (**D**) multivariate Cox regression analyses for TCGA–LUAD. (**E**) ROC curve for PZP protein expression in LUAD clinical samples. (**F**) A comparison was conducted on the OS among patients categorized by their PZP protein expression levels in LUAD clinical samples. (**G**) Univariate and (**H**) multivariate Cox analyses were utilized to examine the relationships between the prognostic characteristics and OS in LUAD clinical samples.

Bioinformatics analysis revealed that the area under the curve (AUC) of the ROC curve was computed to be 0.789 (95% CI: 0.759–0.819) for PZP, indicating a certain level of accuracy (Figure 2A). The survival analysis indicated an evident link between high PZP expression and extended OS, compared to low PZP expression (Cox *p* = 0.007, Figure 2B). Furthermore, univariate analysis (Figure 2C) and multivariate Cox regression analysis (Figure 2D) confirmed PZP as being an independent risk factor. PZP expression was considerably related to the survival probabilities of patients with LUAD assessed at 1 year, 3 years, and 5 years, as demonstrated by the total nomogram points (Figure S2A). The calibration curve assessed the performance of the PZP nomogram, with the C-index (CI) of OS being 0.703 (0.679–0.727) (Figure S2B), which signified the consistency between our nomogram and the actual outcomes.

Next, the effectiveness of the PZP protein in LUAD clinical samples was also analyzed by using an ROC curve, and the AUC was 0.804 (95% CI: CI = 0.747–0.862) (Figure 2E), suggesting that the PZP protein had a high diagnostic value in identifying LUAD. Moreover, all patients were categorized into a PZP-low group (n = 67) and a PZP-high group (n = 43) using the predetermined cutoff value (H-SCROE >40 indicated the PZP-high expression group and ≤40 the PZP-low expression group) by MedCalc software (Version 19.0.4) [30]. PZP expression was correlated with clinical pathological stage but not significantly associated with sex, age, or smoking in our cohort (Table 1). Notably, LUAD tumor tissues exhibiting higher expression of PZP were linked to an elongated OS period, as demonstrated by the Kaplan-Meier analysis (Cox *p* = 0.006, Figure 2F). To validate the predictive value, both univariate and multivariate Cox regression models were employed for analysis. PZP (Cox *p* = 0.001), clinical stage (Cox *p* = 0.001), M-stage (Cox *p* = 0.009) and N-stage (Cox *p* < 0.001) were all recognized as prognostic parameters in the univariate analysis (Figure 2G). In the multivariate Cox regression analysis, PZP continued to be identified as an independent risk factor (Cox *p* = 0.039, Figure 2H). Collectively, these observations indicated that PZP has an unique diagnostic advantage and is remarkably well associated with the prognosis of patients.

### 3.3. PZP Expression Was Associated with TME Modulation in LUAD

**Figure 3:**
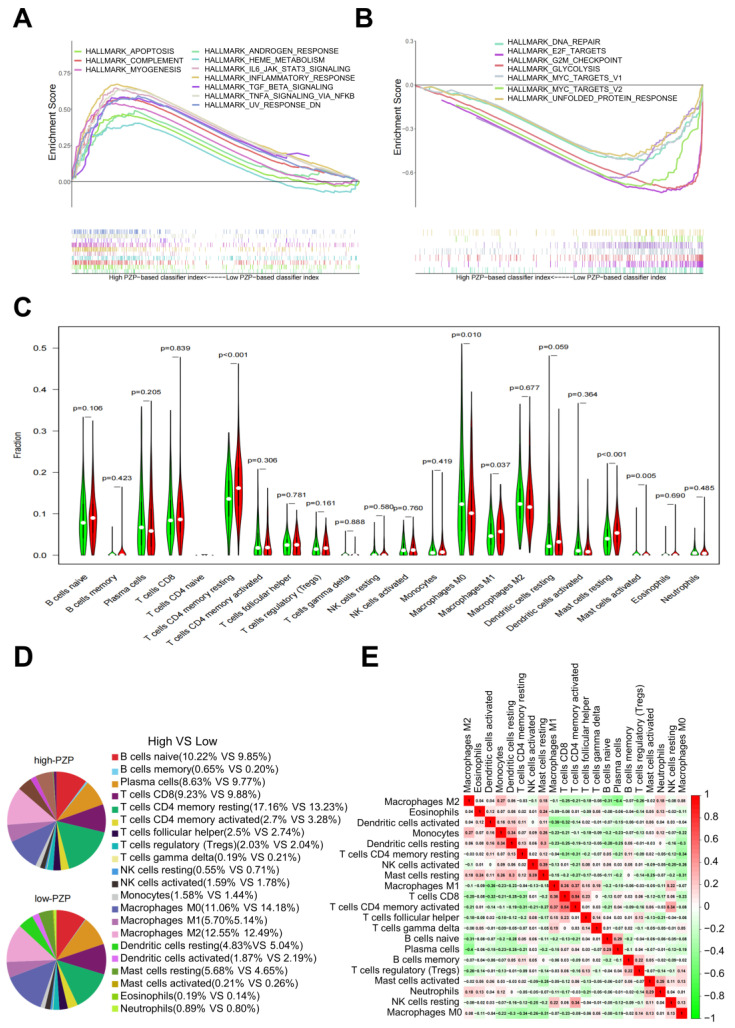
GSEA and CIBERSORT analyzed PZP−related immune cell infiltration alterations in LUAD. (**A**,**B**) PZP was subjected to GSEA, revealing the top 10 pathways that were enriched in the group characterized by high−PZP expression, as well as 7 pathways in the group characterized by low−PZP expression. (**C**) The relative ratio of 22 different immune cell types with varying expression levels of PZP are presented in a series of violin plots. (**D**) A graphical representation of the relative ratio of 22 different immune cell types at different expression levels of PZP was produced. (**E**) Heatmap display of pairwise matrix demonstrating the correlation coefficient values between various immune cell types, based on the Pearson test.

Despite the observed association between abnormal PZP expression and clinicopathological characteristics and OS in patients with LUAD, the exact function of PZP remains unknown. To identify potential signaling pathways perturbed by PZP, the Hallmarks pathway database was plotted using GSEA. It was determined that the genes were primarily enriched in immune-related activities, including inflammatory response, TGF-β signaling, complement, and IL6-JAK-STAT3 signaling pathways in the high-PZP expression group (Figure 3A and Table S1). In contrast, cell metabolism (glycolysis), cancer-related signals (Myctargets, E2F targets), and cell cycle regulation (G2M-checkpoint, DNA repair) were the primary pathways enriched among the genes in the PZP-low expression group (Figure 3B and Table S2).

Tumor-infiltrating immune cells (TIICs) represent a significant component of the tumor microenvironment (TME), and mutual signaling between cancer cells and immune cells can reduce the anticancer activity of endogenous TIICs and promote tumor immune escape. TIICs are closely associated with tumor proliferation, angiogenesis and metastasis; therefore, we explored the correlation between PZP expression and 22 kinds of TIIC profiles in LUAD samples, utilizing the CIBERSORT algorithm. The findings indicated a positive correlation between PZP expression and four types of TIICs, containing resting CD4 memory T cells (Wilcoxon *p* < 0.001), activated mast cells (Wilcoxon *p* = 0.005), resting mast cells (Wilcoxon *p* < 0.001), and M1 macrophages (Wilcoxon *p* = 0.037), as an inverse correlation was observed with M0 macrophages (Wilcoxon *p* = 0.01) (Figure 3C). Among these correlations, the most significant decrease in TIICs in the high-PZP expression group was observed in M0 macrophages, with a percentage of 11.06%, compared to 14.18% in the group characterized by low-PZP expression (Figure 3D). Conversely, the most remarkable increases were exhibited in the following TIIC subtypes: resting CD4 memory T cells (17.24% vs. 13.23%), M1 macrophages (5.77% vs. 5.08%), and resting mast cells (5.68% vs. 4.65%). Moreover, the correlation coefficient was depicted in a heatmap between various TIIC subpopulations, revealing some immune cell types that exhibited correlations in LUAD tissue (Figure 3E). Of note, the correlation analysis revealed a significant positive relationship (Pearson’s r = 0.54) between CD4 memory T cells and activated CD8 T cells, whereas M2 macrophages and plasma cells demonstrated a significant negative correlation, suggesting a strong inverse relationship (Pearson’s r = −0.4). In combination, these findings indicate that PZP assumes an integral role in governing the composition of TIIC subtypes within the TME.

### 3.4. Relationship between PZP and Immune Cells in Clinical Samples

**Figure 4:**
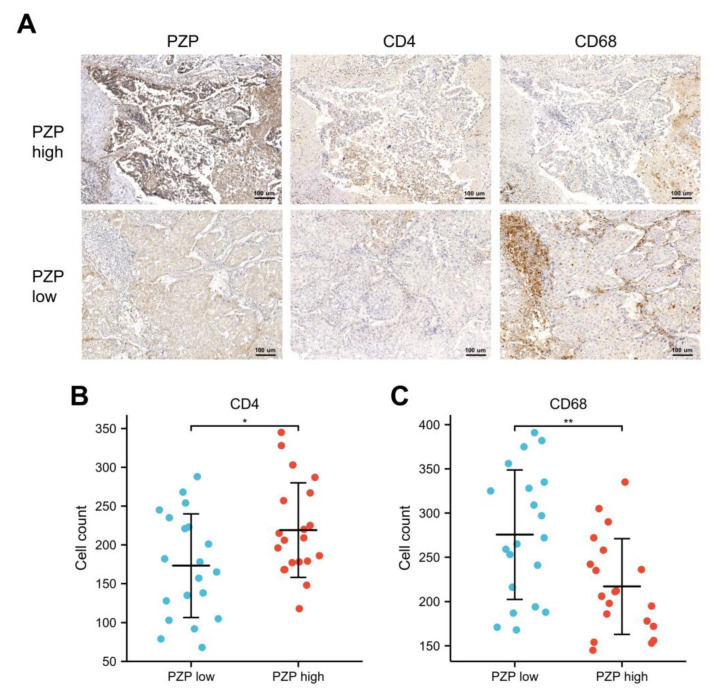
CD4 and CD68 IHC staining was carried out to identify the infiltration of T-cells and macrophages in LUAD tissues characterized by different PZP expression levels. (**A**,**B**) Number of CD4+ T cells infiltrated in the groups characterized by low-PZP expression and high-PZP expression. (**A**,**C**) Number of CD68+ macrophages infiltrated in the groups characterized by low-PZP expression and high-PZP expression. N = 20 for the PZP-low and PZP-high groups. * *p* < 0.05, ** *p* < 0.01.

The degree and proportion of infiltration by immune cells in the TME can have varying impacts on tumor prognosis; for example, macrophages and CD4+ T cells serve important functions in promoting or killing lung cancer [31,32]. To further investigate the link between PZP and immune cell infiltrates, we determined some markers of immune cells by means of CIBERSORT analysis. Notably, a remarkably higher number of CD4+ T cells (118–345, median 219 cells/field) appeared in the high-PZP expression group, compared to the low-PZP expression group (60–288, median 173 cells/field) (Student’s *t*-test *p* = 0.029, Figure 4A,B). Conversely, there was a significantly lower number of CD68+ M0 macrophages (145–335, median 217 cells/field vs. 168–391, median 278 cells/field, Student’s *t*-test *p* = 0.006, Figure 4A,C). These results suggest that PZP is strongly associated with CD4+ T cells and macrophages in LUAD.

### 3.5. Association between PZP and the Polarization State of Macrophages

**Figure 5:**
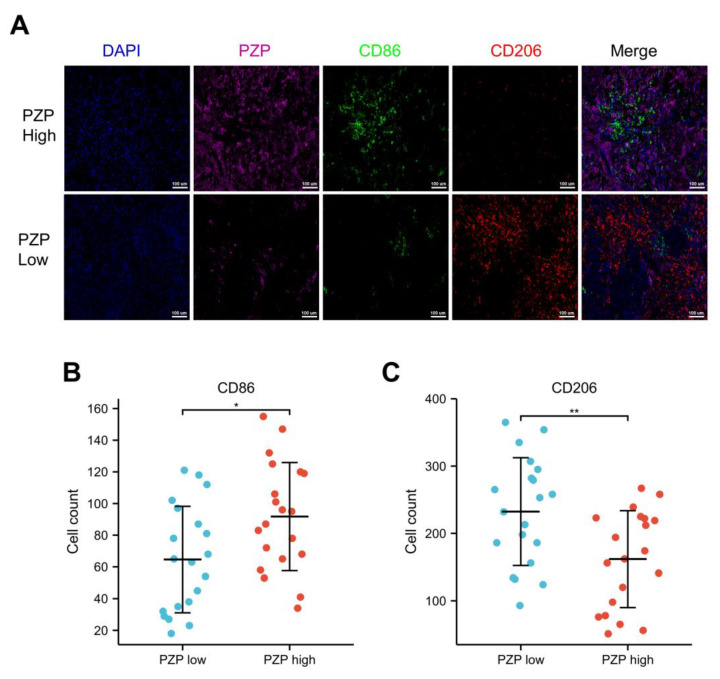
Different expression levels of PZP in LUAD tissues correlated with the presence of polarized M1 and M2 macrophages. (**A**,**B**) The PZP-high and PZP-low expression groups exhibited different counts of infiltrated polarized M1 macrophages, 520 channel for CD86 (FITC, green). (**A**,**C**) Number of polarized M2 macrophages infiltrated in the high-PZP expression and low-PZP expression groups, 570 channel for CD206 (Cy3, red). The 650 channels for PZP (Cy5, magenta), DAPI represents nuclei of the cells (blue). * *p* < 0.05, ** *p* < 0.01.

Macrophages are major components of the TME and are frequently referred to as TAMs. They can polarize into different subtypes in different TMEs, which mainly include two functional subtypes: M1 macrophages, often associated with antitumor signaling, and M2 macrophages, known to exert protumor effects [33]. CD86 is considered to be the canonical surface marker of M1 macrophages, while M2 macrophages express CD206 [34]. The results of mIHC further revealed that the high-PZP expression group had a significantly greater number of infiltrating CD86+ M1s (green, 34–155, median 92 cells/field). In comparison, the low-PZP expression group had a lower number of infiltrating CD86+ M1 macrophages (18–121, median 65 cells/field) (Student’s *t*-test *p* = 0.016, Figure 5A,B). Conversely, the number of CD206+ M2 macrophages (red) was higher in the low-PZP expression group (93–365, median 232 cells/field) than in the high-PZP expression group (51–267, median 162 cells/field) (Student’s *t*-test *p* = 0.006, Figure 5A,C), implying that high PZP expression may have a role in driving macrophage polarization.

## 4. Discussion

LUAD has high morbidity and mortality rates and a poor prognosis, so the demand for diagnostic markers and therapeutic targets with high specificity is critical and time-sensitive. PZP is widely expressed in various cells, tissues and organs, such as in villi [35], the liver [36], breast tissue [13], neutrophils [37] and healthy human plasma exosomes [38]. It plays an important role in maintaining proteostasis during pregnancy [39], inhibiting nerve protrusion extension [40], and is involved in obesity and related metabolic disorders [41]. Nevertheless, the function of PZP in patients with LUAD has not been fully elucidated.

This investigation demonstrated that LUAD tissues exhibit markedly reduced levels of PZP expression compared to noncancerous lung tissues. Moreover, PZP concentration in the supernatant from BEAS-2B cells was notably higher than that in LUAD cell lines. It was also noted that PZP expression was significantly linked to clinical stage, with patients demonstrating greater proportions of low-PZP expression with increasing clinical stage. These discoveries highlight the notion that PZP may function as a tumor-suppressing gene in LUAD. ROC analysis suggested that the PZP protein had a high diagnostic value in distinguishing LUAD. Survival analysis and Cox proportional regression analysis implied that declined PZP expression was found to be positively correlated with an adverse prognosis and that PZP may independently contribute to the risk profile of patients with LUAD. Low expression of PZP was revealed to be related to adverse prognosis in BC and even lower expression in triple-negative BC [13]. PZP is expressed at low levels in hepatocellular carcinoma with poor clinical prognosis and is considered to be a new biomarker to predict the prognosis of hepatocellular carcinoma [14,15,16,17,18]. These findings collectively emphasize the function of PZP expression in assessing survival and its prognostic value as a biomarker for LUAD.

We next used GSEA to determine the mechanism associated with PZP in LUAD. Surprisingly, the investigations revealed that the group characterized by high PZP expression clustered many immunomodulatory pathways, such as complement, UV response, inflammatory response, TGF-β signaling, and IL6-JAK-STAT3 signaling, demonstrating that PZP may influence the progression of LUAD by regulating immune-related molecules in the TME. TIICs are recognized to serve as fundamental components in lung cancer progression and immunity [19,20,21,22]. Consequently, we performed a CIBERSORT analysis to assess whether PZP had an effect on 22 immune cells in LUAD. A significant alteration in the distribution of TIICs was observed between the groups characterized by low-PZP expression and high-PZP expression, with an apparent increase in the proportion of M1 macrophages, resting dendritic cells, resting memory CD4 T cells, resting mast cells, and mast cell-activated cells, while the proportion of M0 macrophage cells markedly decreased. We subsequently confirmed a positive correlation between PZP expression and CD4+ T cells, as well as a negative correlation with CD68+ M0 macrophages, in our clinical samples, suggesting that PZP was likely to influence the progression of LUAD by rebuilding the TME, driving the formation of an immuno-enhancement microenvironment, and modulating TIIC infiltration, including innate immune cells and adoptive immune cells.

The most abundant components of the tumor microenvironment are tumor-associated macrophages, which mainly include M1 and M2 subtypes [33]. Accumulating reports provide evidence that macrophages infiltrated by tumor tissues polarized to M1 and M2 types, which participated in prolonging and decreasing the lung cancer patient’s survival time, respectively [42,43,44]. We, therefore, examined the relationship between PZP and polarized macrophages by means of mIHC. PZP expression showed an evident positive correlation with CD86+ M1 macrophages and an obvious negative correlation with CD206+ M2 macrophages. JENSEN and Gliemann reported that human monocyte-macrophages mediate the binding, uptake, degradation and clearance of the PZP–proteinase complex [11,45], and PZP likely alters the polarization of macrophages by interacting with the receptors LRP1 [40] and GRP78 [41]. Macrophage infiltration and polarization are considered to be important events in the cancer process [46,47]. Polarization of macrophages into M1 results in antitumor efficacy, while polarization into M2 promotes pro-tumor effects [48,49]. Our findings implied an evident correlation between PZP expression and M1 macrophages, enabling speculation that PZP may have affected the infiltration of M1 macrophages and, subsequently, led to improved patient prognosis.

Finally, the limitations of this study were that only a small number of patients were validated. A larger sample needs to be validated, and more investigation is necessary to uncover the specific mechanisms of how PZP regulates TIICs and macrophage polarization in the pathogenesis and progression of LUAD.

## 5. Conclusions

In summary, our observations illustrated that PZP expression was reduced in LUAD tissues and gave rise to poor prognosis in patients. In addition, PZP may be involved in the regulation of TIICs within the landscape of LUAD’s TME, particularly with respect to the infiltration of polarized macrophages. These discoveries indicate that PZP expression could be a useful biomarker for predicting LUAD prognosis and that PZP expression holds great potential promise as an immune-related therapeutic target.

## Figures and Tables

**Table 1:** Correlation between PZP expression and clinicopathologic characteristics of patients with LUAD.

PZP Expression				
Characteristics	NO. of Cases(%)	Low NO.Cases	High NO.Cases	*p* Value
Age (y)				
≤65	46 (41.8%)	28	18	0.994
>65	64 (58.2%)	39	25	
Gender				
Female	59 (53.6%)	33	26	0.250
Male	51 (46.4%)	34	17	
Smoker				
No	63 (57.3%)	37	26	0.588
Yes	47 (42.7%)	30	17	
T classification				
T1&T2	104 (94.5%)	62	42	0.247
T3&T4	6 (5.5%)	5	1	
N classification				
N0	71 (64.5%)	38	33	0.032
N1&N2&N3	39 (35.5%)	29	10	
M classification				
M0	97 (88.2%)	56	41	0.062
M1	13 (11.8%)	11	2	
Clinical stage				
I&II	82 (74.5%)	43	39	0.002
III&IV	28 (25.5%)	24	4	

## Data Availability

The authors confirm that all data supporting the findings of this study will be made available without undue reservation. The results shown here are partly based on data generated by the TCGA Research Network (https://www.cancer.gov/tcga, accessed on 2 November 2020); GTEx (https://www.gtexportal.org/, accessed on 2 November 2020); CIBERSORT (http://cibersort.stanford.edu/, accessed on 2 November 2020); HPA (www.proteinatlas.org, accessed on 11 May 2021); CPTAC (http://ualcan.path.uab.edu/analysis-prot.html, accessed on 11 May 2021); GSEA (http://www.broadinstitute.org/gsea/index.jsp, accessed on 17 February 2022).

## Supplementary Materials

The following supporting information can be downloaded at https://www.mdpi.com/article/10.3390/biomedicines11071978/s1, Figure S1: HPA and CPTAC. (A) The analysis of PZP protein expression between normal and LUAD tissues conducted by HPA. (B) Plotting the statistical data of PZP protein expression between normal and LUAD tissues obtained through CPTAC analysis; Figure S2: The nomogram and calibration curve. (A) A nomogram that integrates PZP and other prognostic factors in LUAD. (B) Charts illustrating the calibration of the nomogram-predicted survival rate and the observed survival rate in calibration plots; Figure S3: Spearman’s correlation analysis between PZP+ tumor cell density and the density of various immune cells in LUAD. R, Spearman coefficient; Table S1: The PCR primers of PZP and GAPDH; Table S2: Gene set associated with high PZP mRNA expression enrichment; Table S3: Gene set associated with low PZP mRNA expression enrichment.
