# Supplementary material for: Pregnancy Zone Protein Serves as a Prognostic Marker and Favors Immune Infiltration in Lung Adenocarcinoma
Source: Biomedicines. 2023 Jul 13;11(7):1978. doi: 10.3390/biomedicines11071978 (PMC10377424; doi:10.3390/biomedicines11071978)
Supplement: Supplementary file 1 [file biomedicines-11-01978-s001.zip › biomedicines-2472455-supplementary.pdf]

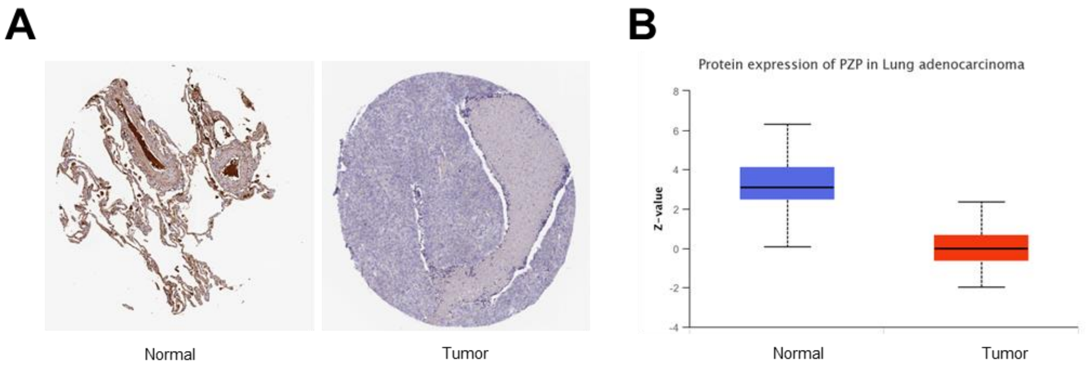

Figure S1. HPA and CPTAC. (A) The analysis of PZP protein expression between normal and LUAD tissues conducted by HPA. (B) Plotting the statistical data of PZP protein expression between normal and LUAD tissues obtained through CPTAC analysis. (Normal=111, Tumor=111, Student's t-test p ＜ 0.001).

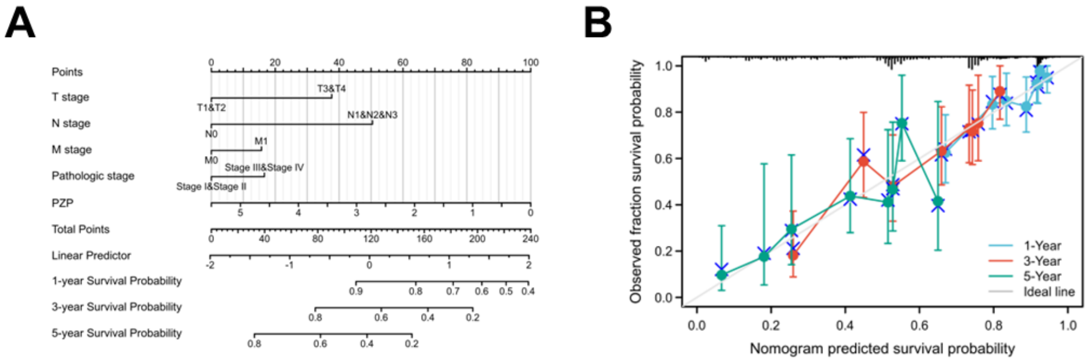

Figure S2. The nomogram and calibration curve. (A) A nomogram that integrates PZP and other prognostic factors in LUAD. (B) Charts illustrating the calibration of the nomogram-predicted survival rate and the observed survival rate in calibration plots. Actual survival is shown on the Y axis, and nomogram-predicted survival is shown on the X axis.

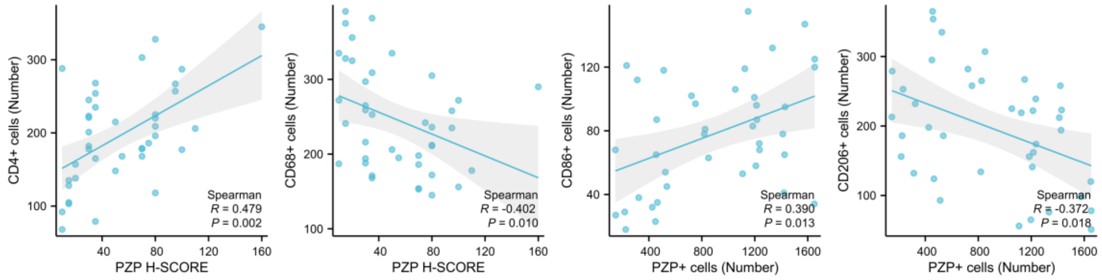

Figure S3. Spearman's correlation analysis between PZP+ tumor cell density and the density of various immune cells in LUAD. R, Spearman coefficient.

Table S1. The PCR primers of PZP and GAPDH.

Gene	Sense Primer (5'-3')	Antisense Primer (5'-3')
PZP	GGACCAGCAGGAGGAAGAAC	GGATGACTGAACACGACTTTGG
GAPDH	GCACCGTCAAGGCTGAGAAC	TGGTGAAGACGCCAGTGGA

Table S2. Gene set associated with high PZP mRNA expression enrichment.

NAME	SIZE	ES	NES	NOM.p.val	FDR.q.val
HALLMARK_COMPLEMENT	200	0.5820853	1.7799287	0.006072875	0.09347308
HALLMARK_UV_RESPONSE_DN	144	0.5929021	1.7730963	0.00409836	0.052168243
HALLMARK_TGF_BETA_SIGNALING	54	0.5841645	1.7475767	0.018867925	0.05012179
HALLMARK_ANDROGEN_RESPONSE	100	0.49081752	1.7442752	0.008032128	0.040799465
HALLMARK_TNFA_SIGNALING_VIA_NFKB	200	0.64434105	1.7027507	0.011857707	0.05716673
HALLMARK_INFLAMMATORY_RESPONSE	200	0.67309904	1.7009398	0.01039501	0.047638945
HALLMARK_IL6_JAK_STAT3_SIGNALING	87	0.6473242	1.6452171	0.02892562	0.08073055
HALLMARK_HEME_METABOLISM	199	0.4047689	1.6247531	0.002016129	0.08633792
HALLMARK_MYOGENESIS	200	0.54191417	1.6226106	0.010706638	0.07771326
HALLMARK_APOPTOSIS	161	0.46726292	1.5917807	0.026156941	0.09338874
HALLMARK_KRAS_SIGNALING_UP	200	0.55152625	1.5916247	0.032786883	0.08489886
HALLMARK_COAGULATION	138	0.5132748	1.5713077	0.018867925	0.09090983
HALLMARK_APICAL_SURFACE	44	0.5725144	1.5650676	0.016877636	0.08761066
HALLMARK_IL2_STAT5_SIGNALING	199	0.5087707	1.5445342	0.03797468	0.09624986
HALLMARK_XENOBIOTIC_METABOLISM	200	0.44930765	1.5354816	0.017857144	0.0961352
HALLMARK_BILE_ACID_METABOLISM	112	0.4690251	1.5173465	0.025052192	0.10140155
HALLMARK_ESTROGEN_RESPONSE_EARLY	200	0.42526257	1.4248351	0.02972399	0.13692656
HALLMARK_KRAS_SIGNALING_DN	200	0.43439096	1.4077725	0.004246285	0.14535348

Table S3. Gene set associated with low PZP mRNA expression enrichment.

NAME	SIZE	ES	NES	NOM.p.val	FDR.q.val
HALLMARK_MYC_TARGETS_V1	200	-0.640592	-1.8738643	0	0.007630331
HALLMARK_DNA_REPAIR	150	-0.5222676	-1.7626995	0	0.02247388
HALLMARK_UNFOLDED_PROTEIN_RESPONSE	113	-0.4966947	-1.7418033	0.006224067	0.01827278
HALLMARK_G2M_CHECKPOINT	200	-0.7138411	-1.7054588	0.006060606	0.02177113
HALLMARK_MYC_TARGETS_V2	58	-0.70146245	-1.6990482	0.006198347	0.018629335
HALLMARK_E2F_TARGETS	200	-0.7395699	-1.6715649	0.006036217	0.022106228
HALLMARK_GLYCOLYSIS	200	-0.51202047	-1.6206344	0.008474576	0.03119186
